# The Egyptian wheat cultivar Gemmeiza-12 is a source of resistance against the fungus *Zymoseptoria tritici*

**DOI:** 10.1186/s12870-024-04930-y

**Published:** 2024-04-05

**Authors:** Abdelrahman M Qutb, Florence Cambon, Megan C McDonald, Cyrille Saintenac, Graeme J Kettles

**Affiliations:** 1 School of Biosciences, University of Birmingham, Edgbaston, Birmingham, B15 2TT UK; 2 Department of Agricultural Botany, Faculty of Agriculture, Al-Azhar University, Cairo, Egypt; 3 Université Clermont Auvergne, INRAE, GDEC, Clermont-Ferrand, 63000 France

**Keywords:** *Zymoseptoria tritici*, Wheat, Disease resistance, *Stb6*, Stomata

## Abstract

**Background:**

Wheat is one of the world’s most important cereal crops. However, the fungal pathogen *Zymoseptoria tritici* can cause disease epidemics, leading to reduced yields. With climate change and development of new agricultural areas with suitable environments, *Z. tritici* may advance into geographical areas previously unaffected by this pathogen. It is currently unknown how Egyptian wheat will perform in the face of this incoming threat. This project aimed to assess the resistance of Egyptian wheat germplasm to *Z. tritici*, to identify cultivars with high levels of resistance and characterise the mechanism(s) of resistance present in these cultivars.

**Results:**

Eighteen Egyptian wheat cultivars were screened against two *Z. tritici* model isolates and exhibited a wide spectrum of responses. This ranged from resistance to complete susceptibility to one or both isolates tested. The most highly resistant cultivars from the initial screen were then tested under two environmental conditions against modern UK field isolates. Disease levels under UK-like conditions were higher, however, symptom development on the cultivar Gemmeiza-12 was noticeably slower than on other Egyptian wheats. The robustness of the resistance shown by Gemmeiza-12 was confirmed in experiments mimicking Egyptian environmental conditions, where degree of *Z. tritici* infection was lower. The Kompetitive allele-specific PCR (KASP) diagnostic assay suggested the presence of an *Stb6* resistant allele in several Egyptian wheats including Gemmeiza-12. Infection assays using the IPO323 WT and IPO323Δ*AvrStb6* mutant confirmed the presence of *Stb6* in several Egyptian cultivars including Gemmeiza-12. Confocal fluorescence microscopy demonstrated that growth of the IPO323 strain is blocked at the point of stomatal penetration on Gemmeiza-12, consistent with previous reports of *Stb* gene mediated resistance. In addition to this *R*-gene mediated resistance, IPO323 spores showed lower adherence to leaves of Gemmeiza-12 compared to UK wheat varieties, suggesting other aspects of leaf physiology may also contribute to the resistance phenotype of this cultivar.

**Conclusion:**

These results indicate that Gemmeiza-12 will be useful in future breeding programs where improved resistance to *Z. tritici* is a priority.

**Supplementary Information:**

The online version contains supplementary material available at 10.1186/s12870-024-04930-y.

## Introduction

Wheat is one of the most widely-grown cereal crops, and along with maize and rice, contributes significantly to global food security [1]. Of the estimated ∼130 million farms worldwide, a fifth are dedicated to growing wheat [2] and worldwide wheat production in season 2020–2021 was 772.64 million metric tonnes (MMT) [3]. In the UK, from 2015 to 2019 wheat production accounted for 68.5% of the value of the cereals sector, amounting to approximately £2.4 billion. This figure represents roughly one-quarter of the total contribution of agriculture to the UK economy [4]. Wheat also holds significant importance as the primary cereal crop in Egypt, contributing ∼ 10% of the total value of national agricultural production. Despite its significance in domestic agriculture, Egypt remains the world’s largest importer of wheat [5], with around 50% of the consumed wheat being imported annually [6]. As a result, securing wheat productivity is of crucial importance for Egyptian agriculture.

Septoria tritici blotch (STB) is a foliar disease of wheat caused by the fungus *Zymoseptoria tritici*. It is a significant disease threat in many wheat-producing regions and has been reported in South America, Africa, Asia, North America, Europe and Oceania [7]. In addition, STB leads to substantial yield losses in countries near Egypt, such as Tunisia, Morocco, Greece, Turkey, Algeria, and Ethiopia [8–13]. Due to climate change, it is anticipated that the expansion of pathogen ranges into previously unfavourable geographical regions will occur [14]. For example, a recent study suggests that rising temperatures have contributed to the escalation of STB epidemics, resulting in a decline in wheat production across Germany [15]. A study conducted in 2018 projected that future wheat yield will decline due to the negative impacts of climate change such as the expansion of the pathogen range [16]. However, no previous studies have investigated the performance of Egyptian wheat varieties exposed to *Z. tritici*. Climate change and the development of new wheat-growing areas with more favourable conditions for the fungus may therefore present a future challenge of STB in Egypt.

*Z. tritici* is a filamentous fungus belonging to the Dothideomycetes class and can produce both sexual ascospores and asexual pycnidiospores during wheat infections [17, 18]. Infection by *Z. tritici* begins when pycnidiospores and ascospores land on the surface of wheat leaves, subsequently germinating and forming invasive hyphae that penetrate the leaf tissue through openings such as stomata [19]. Following this, the fungus colonises the apoplastic spaces within the leaf mesophyll without inducing significant immune responses or macroscopic disease symptoms [17, 20, 21]. Subsequently, the fungus transitions into the necrotrophic growth phase, marked by reproductive processes in the later stages of infection [22]. The symptoms of STB manifest as chlorotic patches, which gradually enlarge into light-brown necrotic lesions harbouring darker-coloured fruiting bodies [23].

Wheat exhibits two forms of resistance to STB: qualitative and quantitative resistance [24]. Qualitative resistance is governed by disease *resistance* (*R*) genes known as *Stb* genes, which appear to operate in a gene-for-gene manner [25] This type of resistance typically exerts a potent effect but can be overcome by mutations in pathogen avirulence (*Avr*) genes. To date, twenty three *Stb* genes have been identified within the wheat genome [26–28]. In contrast, quantitative resistance is determined by multiple quantitative trait loci (QTLs) and allows for some disease progression but is more challenging for the pathogen to overcome [29]. Several *Stb* genes that provide qualitative resistance have recently been cloned. *Stb6*, a common resistance gene in European germplasm [24], encodes a wall-associated receptor kinase (WAK)-like protein [30] that confers resistance to *Z. tritici* isolate IPO323 which has the corresponding avirulence gene *AvrStb6* [31]. *Stb16q* was demonstrated to encode a plasma membrane cysteine-rich receptor-like kinase that significantly inhibits pathogen growth and penetration in cultivated wheat [32]. However, virulence to *Stb16q* has recently been detected in Irish [33] and French *Z. tritici* populations [34]. More recently, *Stb15* has been shown to encode a cell-surface lectin receptor-like kinase, that can lead to complete resistance [35]. *Stb15* plays a significant role in resistance against the isolate IPO88004 and is noteworthy for its prevalence in European wheat cultivars [35].

To date, no study has investigated the level of resistance to *Z. tritici* present in Egyptian wheat germplasm. This study is the first to assess the relative susceptibility of Egyptian wheat cultivars to *Z. tritici*. Our aims are to (i) identify the best sources of resistance against this pathogen present in locally-adapted Egyptian wheat, and (ii) characterise the resistance mechanism(s) of the most promising varieties.

## Results

### Variable STB resistance exists within Egyptian wheat germplasm

**Fig. 1:**
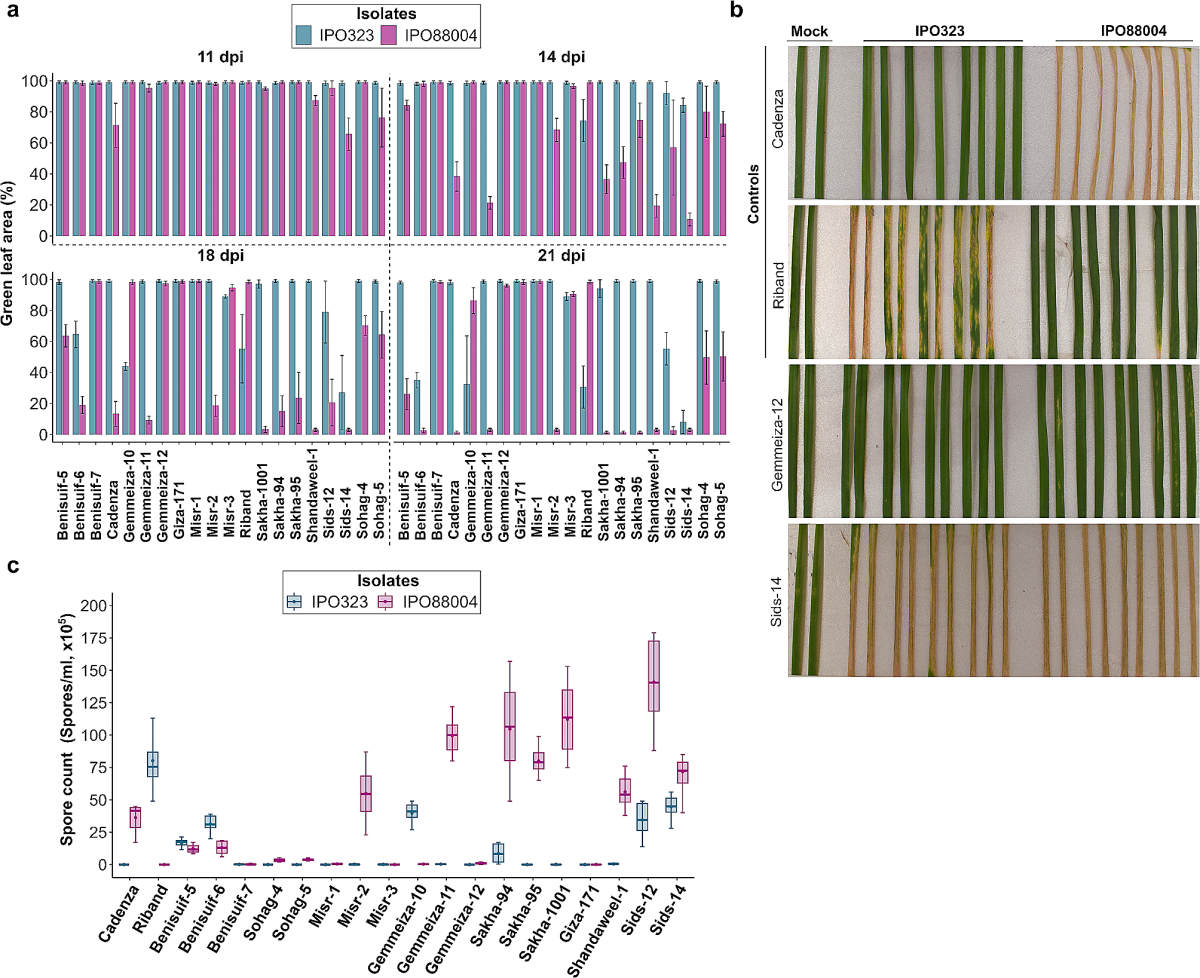
Egyptian wheat has variable resistance against *Z. tritici*. **(a)** The resistance of 18 Egyptian wheat cultivars was tested against IPO323 and IPO88004 and % green leaf area (GLA) was assessed. Cadenza and Riband were used as controls. The data was analysed using an image analysis software MIPAR. Leaves assessed at 11,14,18, and 21 dpi. Mean and standard deviations were calculated with data from nine independent replicates. **(b)** After 21 dpi, Gemmeiza-12 showed high resistance to IPO323 and IPO88004. **(c)** Pycnidiospores were washed from leaves at 21 dpi. Mean and standard deviations were calculated with data from eight independent replicates

To assess the level of disease resistance present in Egyptian wheat germplasm, 18 cultivars were screened under controlled environmental conditions. These 18 cultivars are currently amongst the most widely grown wheat cultivars in Egypt [36]. According to the Egyptian Economic Affairs Sector (https://www.agri.gov.eg/library/25), in 2021 these 18 cultivars were grown on 87.2% of the total wheat-grown area in Egypt (Additional file 1). The UK winter wheat cultivar Riband was used as a control as it is highly susceptible to the *Z. tritici* isolate IPO323 but resistant to IPO88004. In contrast, the UK spring wheat cultivar Cadenza is highly resistant to IPO323 but susceptible to IPO88004. On these control varieties, disease symptoms began appearing at approximately 12 days post inoculation (dpi) and were visible as chlorotic patches before transition to necrosis (Fig. 1). The development of disease symptoms varied among the Egyptian wheat cultivars during IPO88004 infection. Three different responses were observed in the 18 Egyptian wheat cultivars exposed to IPO88004, high resistance, moderate susceptibility, and high susceptibility. The group of highly resistant cultivars demonstrated similar resistance to IPO88004 as the control cultivar Riband up to 21 dpi. These were Misr-1, Misr-3, Gemmeiza-12, Giza-171, and Benisuif-7 (Fig. 1a). The group of moderately susceptible cultivars included Sohag-4, Sohag-5, and Benisuif-5 (Fig. 1a). The third group of highly susceptible cultivars that demonstrated similar susceptibility to IPO88004 as the Cadenza control were Shandaweel-1, Sakha-94, Sakha-1001, Sakha-95, and Gemmeiza-11 (Fig. 1a). Of the Egyptian cultivars, Gemmeiza-12 remained symptomless at 21 dpi, similar to the Riband control leaves (Fig. 1b). In contrast, complete necrosis and fungal sporulation was observed on leaves of Cadenza and Sids-14 (Fig. 1b). Two different responses were observed in the Egyptian cultivars exposed to IPO323, high resistance or high susceptibility (Fig. 1a). The first group comprised highly resistant cultivars, with 15/18 Egyptian cultivars maintaining a mean green leaf area (GLA) of > 50% at 21 dpi. In contrast, the second group contained three highly susceptible cultivars that demonstrated similar susceptibility to IPO323 as Riband by 21 dpi. These were Benisuif-6, Sids-14, Gemmeiza-10, and Sids-12.

### IPO323 and IPO88004 failed to sporulate on highly resistant Egyptian cultivars

To test whether the variable levels of resistance observed by measuring GLA translated to level of fungal sporulation, spore washes were collected from leaves of all cultivars. On cultivars that were highly resistant to both *Z. tritici* isolates, namely Misr-1, Misr-3, Gemmeiza-12, Giza-171, and Benisuif-7, neither isolate was able to sporulate, as demonstrated by the extremely low numbers of spores observed in leaf washes (Fig. 1c). However, IPO88004 did successfully sporulate on Sids-14, Sids-12, Shandaweel-1, Sakha-1001, Sakha-95, Sakha-94, Gemmeiza-11, and Misr-2 (Fig. 1c). This was consistent with the level of resistance as demonstrated by measurement of GLA (Fig. 1a). In comparison, IPO323 only sporulated on cultivars Sids-12, Sids-14, Gemmeiza-10, and Benisuif-6. Again, this was in agreement with the level of resistance as measured by GLA. Additionally, IPO88004 and IPO323 were able to successfully sporulate on either of the controls Cadenza and Riband, consistent with GLA measurements.

### Gemmeiza-12 is the most resistant Egyptian hexaploid cultivar to modern *Z. tritici* isolates

**Fig. 2:**
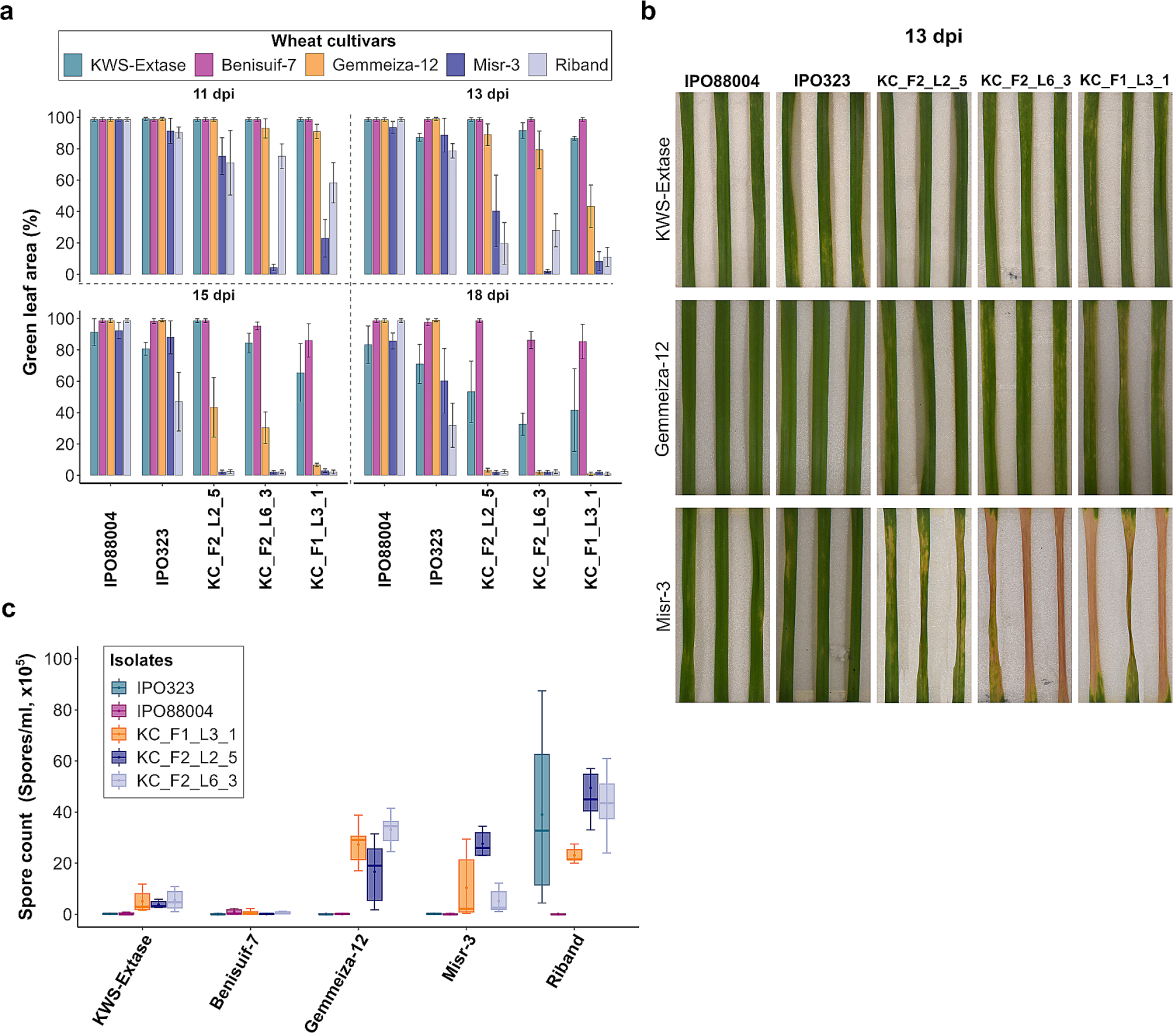
Egyptian wheat is susceptible to several modern UK *Z. tritici* isolates under UK-like conditions. **(a)** Disease development of three modern UK *Z. tritici* isolates on Gemmeiza-12, Misr-3, and Benisuif-7. KWS Extase and Riband were controls. The data was analysed using MIPAR. Leaves assessed at 11,13,15, and 18 dpi. Mean and standard deviations were calculated with data from six independent replicates. **(b)** STB disease development on *Z. tritici* isolates at 13 dpi. **(c)** Pycnidiospores were washed from leaves at 21 dpi. Mean and standard deviations were calculated with data from six independent replicates

In our initial screen, we utilised two historical *Z. tritici* isolates to examine the level of resistance to this pathogen. However, these isolates are not representative of present day *Z. tritici* populations. To assess whether resistant varieties were also immune to modern fungal strains, we performed a second screen using three isolates recovered from UK wheat fields in 2021. The three tested isolates were selected from a panel of 30 modern UK *Z. tritici* isolates based on their different phenotypes on PDA medium (Additional file 2). We also included the UK winter wheat cultivar KWS-Extase as an additional control, as it displays high resistance to *Z. tritici* in UK field trials. In this experiment, KWS-Extase demonstrated strong resistance against three modern UK *Z. tritici* isolates, as observed through both GLA and spore washes (Fig. 2a,c). The GLA results indicated a notable difference in disease development of the three UK isolates on KWS-Extase and Gemmeiza-12 after 18 dpi. On average, disease development was 53%, 33%, and 41% slower on KWS-Extase compared to Gemmeiza-12 (Fig. 2a). Furthermore, the results revealed that disease development of the same isolates on Gemmeiza-12 was 52%, 45%, and 9% slower than on Misr-3 at 13 dpi (Fig. 2a,b). However, by 18 dpi all inoculated leaves of Gemmeiza-12 and Misr-3 exhibited complete necrosis. The ability of the modern UK *Z. tritici* isolates to sporulate on Gemmeiza-12 and Misr-3 was comparable (Fig. 2c).

**Fig. 3:**
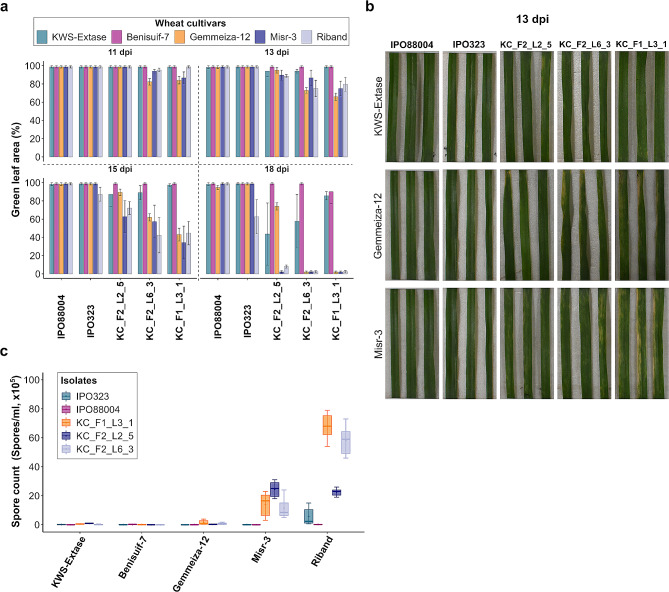
Egyptian wheat is partially resistant to several modern UK *Z. tritici* isolates under Egyptian-like conditions. **(a)** Disease development of three modern UK *Z. tritici* isolates on Gemmeiza-12, Misr-3, and Benisuif-7. KWS Extase and Riband were controls. The data was analysed using MIPAR. Leaves assessed at 11,13,15, and 18 dpi. Mean and standard deviations were calculated with data from six independent replicates. **(b)** STB disease development on *Z. tritici* isolates at 13 dpi. **(c)** Pycnidiospores were washed from leaves at 21 dpi. Mean and standard deviations were calculated with data from six independent replicates

According to the Egyptian Meteorological Authority, the average relative humidity in the Nile Delta of Egypt ranges from 40 to 60%. This contrasts with our controlled environment pathoassay where humidity is typically > 90%. Therefore, to mimic the humidity of Egyptian growing conditions, we reduced the humidity to 50% and repeated these infections (Fig. 3). Under these Egyptian-like conditions, we found that the UK *Z. tritici* isolates still managed to infect both Gemmeiza-12 and Misr-3 cultivars, although the rate of disease development was much slower than in previous experiments. Disease development induced by the three UK isolates on Gemmeiza-12 under Egyptian-like conditions was 6%, 4%, and 23% slower than under the UK-like conditions (Fig. 3a,b). Moreover, disease development of the UK isolates on Misr-3 under the Egyptian-like conditions was 49%, 85%, and 66% slower than under the UK-like conditions. The UK isolates also failed to sporulate on Gemmeiza-12, similar to the KWS-Extase control, although some sporulation was observed on Misr-3 (Fig. 3c). Benisuif-7, the Egyptian durum wheat, exhibited strong resistance against all three UK *Z. tritici* isolates under both environmental conditions (Figs. 2 and 3). This is consistent with the known inability of bread wheat-adapted *Z. tritici* genotypes to infect durum wheat [37].

### *Stb6*, but not *Stb15* or *Stb16q*, contributes to resistance of Gemmeiza-12 and other Egyptian wheat cultivars

We next chose to investigate the contribution of genetic resistance provided by qualitative *R* genes to Gemmeiza-12 and the other Egyptian wheats. As most Egyptian wheats demonstrated resistance to IPO323, which carries an avirulent allele of *AvrStb6*, we utilised the KASP diagnostic assay to test for the presence of resistance-conferring forms of *Stb6*. This analysis suggests that 11/18 Egyptian wheat cultivars possessed the resistant *Stb6* allele, haplotype 1, similar to the positive control Cadenza (Table 1). However, three cultivars (Benisuif-6, Sids-12, Riband) exhibited the susceptible allele, represented by haplotype 7. Additionally, Sids-14 was identified as carrying the susceptible allele, represented by haplotype 3 (Table 1). Notably, several cultivars including Benisuif-5, Sohag-4, Misr-1, Misr-3, and Sakha-94 did not exhibit any of the three haplotypes. This suggests that *Stb*6 may either be absent in these cultivars or present with different, as yet uncharacterised haplotypes (Table 1). Further KASP and PCR assays indicated the absence of the resistant alleles of both *Stb15* (Additional file 3) and *Stb16q* (Additional file 4) in any of the Egyptian wheat cultivars.

**Table 1:** Resistance alleles of Stb6 are detected in some Egyptian wheat cultivars. The *Stb6* resistant allele is represented by haplotype 1. Susceptible alleles are represented by haplotypes 3 and 7. “ND” (not determined) is used when the tested cultivar doesn’t exhibit any of the three tested Stb6 haplotypes

Wheat cultivar	Stb6 Haplotype	Stb6 Allele
Cadenza	1	Resistant
Riband	7	Susceptible
Benisuif-5	ND	Unknown
Benisuif-6	7	Susceptible
Benisuif-7	1	Resistant
Sohag-4	ND	Unknown
Sohag-5	1	Resistant
Misr-1	ND	Unknown
Misr-2	1	Resistant
Misr-3	ND	Unknown
Gemmeiza-10	1	Resistant
Gemmeiza-11	1	Resistant
Gemmeiza-12	1	Resistant
Sakha-94	ND	Unknown
Sakha-95	1	Resistant
Sakha-1001	1	Resistant
Giza-171	1	Resistant
Shandaweel-1	1	Resistant
Sids-12	7	Susceptible
Sids-14	3	Susceptible

**Fig. 4:**
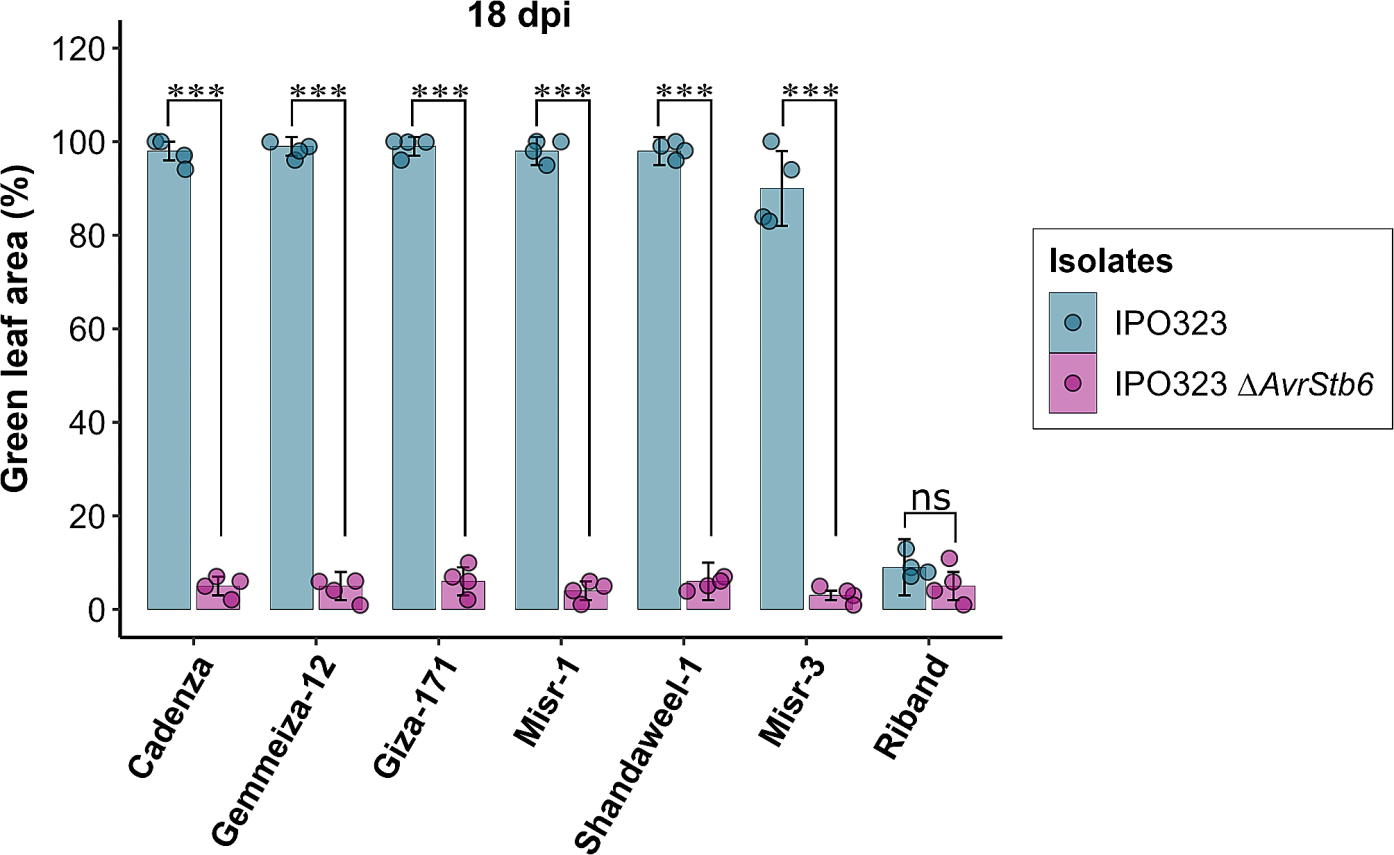
The *Stb6* resistance gene is present in Egyptian wheat. Infection assay with IPO323*∆AvrStb6* strain + WT control. Cadenza and Riband were used as controls. The mean and standard deviation were calculated with data from four independent replicates. Results were analysed for statistical significance using two-way ANOVA with a Tukey post hoc test (***p < 0.001, ns = not significant)

To confirm the role of *Stb6* in conferring resistance to IPO323 in Egyptian wheat, we performed infection assays with wild-type IPO323 and the IPO323*∆AvrStb6* deletion mutant. After 18 dpi, there was a significant difference in GLA between IPO323 and IPO323*∆AvrStb6* infected leaves on all of the tested Egyptian cultivars including Gemmeiza-12 (*P* < 0.001, Tukey Post Hoc; Fig. 4). This confirms our previous genotyping experiments and indicates that resistance of these varieties to IPO323 is indeed due to *Stb6*. In contrast, there was no significant difference in GLA between IPO323 and IPO323*∆AvrStb6* infections on Riband where *Stb6*-based resistance is absent (*P* > 0.05, Tukey Post Hoc; Fig. 4). Together, this indicates that resistant alleles of *Stb6* are present in Gemmeiza-12 and other Egyptian wheats, and that this confers resistance to fungal isolates that are recognised by this *R* gene.

### *Z. tritici* spores have lower adherence to leaves of Gemmeiza-12

**Fig. 5:**
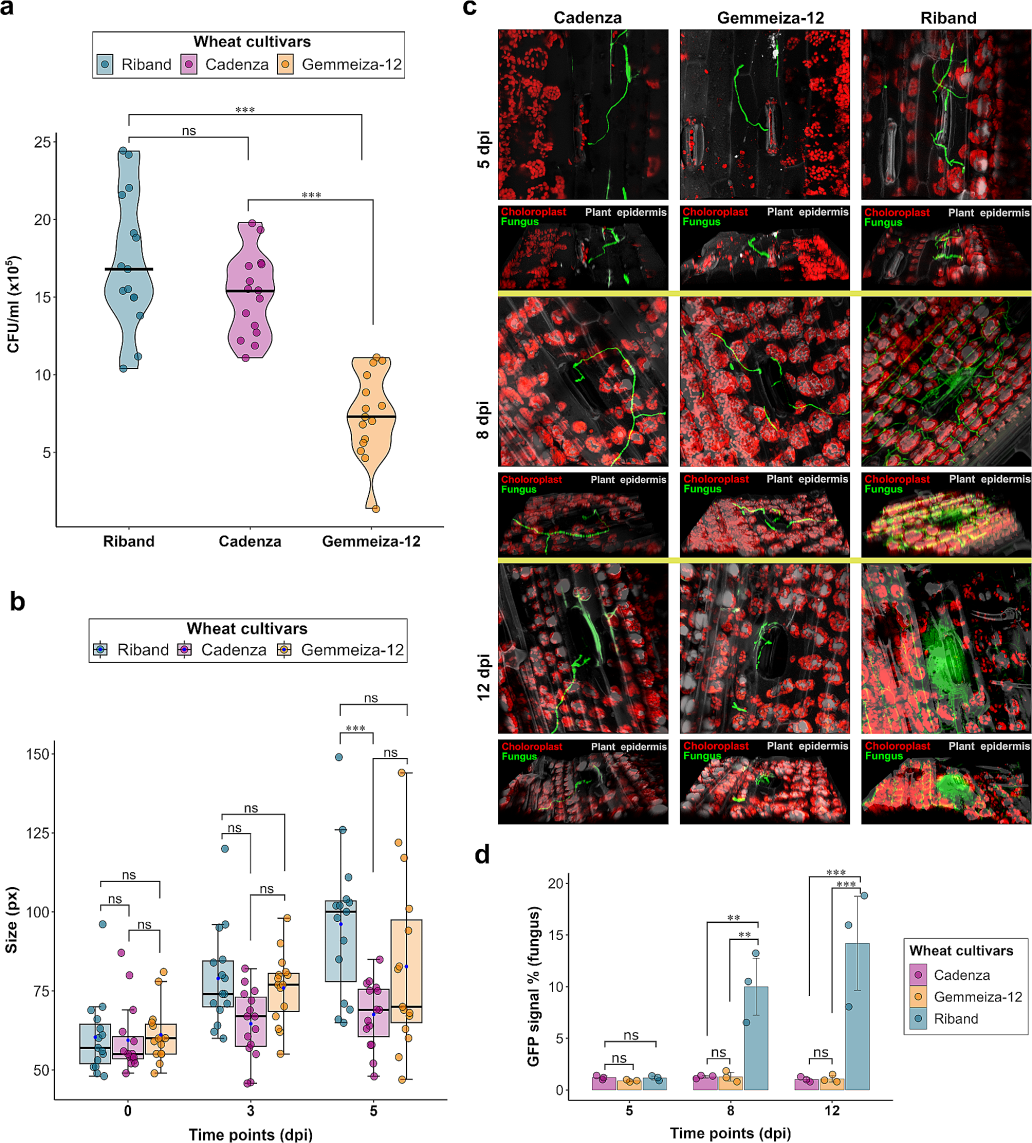
Gemmeiza-12 shows reduced spore adhesion and stomatal penetration. **(a)** Quantitative analysis of IPO323_CZtGFP spore adhesion. The mean was calculated with data from fifteen independent replicates. Results were analysed for statistical significance using one-way ANOVA with a Tukey post hoc test (***p < 0.001; ns = not significant). **(b)** Quantitative analysis of epiphytic fungal growth size (in pixels) of IPO323_CZtGFP. The mean and standard deviation were calculated from fifteen independent replicates. The median values are represented in a blue dot. Results were analysed for statistical significance using two-way ANOVA with a Tukey post hoc test (***p < 0.001; ns = not significant). **(c)** Confocal z-stack images were taken for Cadenza, Gemmeiza-12, and Riband at 5, 8, and 12 dpi. The upper images show a top view, and the lower images show a side view. The fungus, plant chloroplasts, and plant epidermis are depicted in green, red, and grey colours, respectively. Confocal images were processed using Aviris software. **(d)** Quantitative analysis of the fungal GFP signal as % of total signal. The mean and standard deviations were calculated with data from three independent replicates. Results were analysed for statistical significance using two-way ANOVA with a Tukey post hoc test (***p < 0.001, **p < 0.01, ns = not significant)

To initiate infection, *Z. tritici* spores must land on and successfully adhere to wheat leaf surfaces before germinating and penetrating leaves through open stomata. To assess whether these processes are impaired on Gemmeiza-12 plants, we used a fluorescent reporter strain to assess spore adherence, epiphytic growth and stomatal penetration. Firstly, we assessed the adhesion ability of IPO323_CZtGF spores to the leaf surface of Gemmeiza-12 alongside Riband and Cadenza controls. This experiment indicated that IPO323 spores exhibit a significantly lower ability to adhere to the leaf surface of Gemmeiza-12 when compared to Riband and Cadenza (*P* < 0.001, Tukey Post Hoc; Fig. 5a). This indicates that inability of spores to adhere to leaves may contribute to the resistance phenotype of Gemmeiza-12, in addition to the presence of *Stb6*.

### *Z. tritici* failed to colonise the sub-stomatal cavity of Gemmeiza-12

Secondly, we investigated the extent of epiphytic growth of IPO323_CZtGF on Gemmeiza-12 in comparison to Riband and Cadenza. The results showed that there was no significant differences in the extent of growth on the leaf surface of IPO323_CZtGF following germination on the three tested cultivars after 0 and 3 dpi (*P* > 0.05, Tukey Post Hoc; Fig. 5b). At 5 dpi, there was no significant difference in the extent of IPO323_CZtGFP growth on Gemmeiza-12 and Riband (*P* > 0.05, Tukey Post Hoc; Fig. 5b). However, IPO323_CZtGFP growth on Riband was significantly greater than on Cadenza (*P* < 0.001, Tukey Post Hoc; Fig. 5b). Finally, we used the same reporter strain to assess the ability of IPO323_CZtGFP to invade the leaf mesophyll of the same cultivars. Z-stack images captured at three different time points revealed that by 5 dpi, hyphae interacted with and attempted to penetrate the stomata of Gemmeiza-12, Riband, and Cadenza (Fig. 5c; Video 1). The quantitative analysis of z-stack images after 5 dpi showed that there was no significant difference in the fungal green fluorescent protein (GFP) signal among the three tested cultivars (*P* > 0.05, Tukey Post Hoc; Fig. 5d). After 8 dpi, IPO323_CZtGFP failed to invade the stomata in both Gemmeiza-12 and Cadenza but succeeded in invading stomata in Riband and colonising the sub-stomatal cavity (Fig. 5c; Video 1). This led to the formation of fruiting bodies by 12 dpi (Fig. 5c; Video 1). The quantitative analysis of z-stack images after 8 and 12 dpi showed that the fungal GFP signal on Riband was significantly greater than on Gemmeiza-12 and Cadenza (*P* < 0.001, Tukey Post Hoc; Fig. 5d). This indicates that in Gemmeiza-12, similar to Cadenza, invading hyphae are stopped at the point of stomatal penetration by as yet undefined mechanisms.

## Discussion

In this study, an initial screening experiment was conducted using 18 Egyptian commercial wheat cultivars sourced from five breeding stations across Egypt [38]. These cultivars were screened against two model *Z. tritici* isolates IPO323 and IPO88004. In previous studies, it was clearly demonstrated that necrosis and pycnidia formation are governed by distinct genetic loci [39]. Both these traits play essential roles in promoting infection, but the pathogen’s ability to produce spores on a particular cultivar significantly contributes to more widespread and severe field epidemics [40]. Another previous study showed that *Z. tritici* isolates WAI332, WAI323, and WAI321 induced necrotic lesions on the leaves of the wheat cultivar WW2449 [41]. However, notable variations were observed in the quantity of pycnidia generated by each isolate. In this study, we employed two distinct scoring methods to assess the disease virulence of *Z. tritici* isolates during the screening experiment. The first approach involved evaluating the necrotic lesions on wheat leaves using a GLA quantitative scoring method based on GLA analysis. The second approach involved spore counting at 21 dpi to assess the ability of the *Z. tritici* isolates to sporulate on different wheat cultivars. The outcomes of the initial screening of the 18 Egyptian wheat cultivars against IPO323 and IPO88004 revealed a diverse spectrum of responses. We observed cultivars ranging from being highly susceptible to both isolates to being completely resistant to both. Notably, none of the screened Egyptian cultivars were bred with specific resistance to STB disease. Instead, their breeding focus was primarily directed towards acquiring resistance against other diseases, such as rust diseases [38, 42–45]. It was observed that the majority of the Egyptian wheat cultivars exhibited resistance to IPO323, while remaining susceptible to IPO88004. However, five specific cultivars, namely Gemmeiza-12, Misr-1, Misr-3, Giza-171, and Benisuif-7, demonstrated resistance against both IPO323 and IPO88004, as evidenced by the failure of both isolates to induce necrosis or sporulate on these cultivars.

Considering that both IPO323 and IPO88004 are historical isolates obtained from field samples in the last century [46, 47], and given that the present day population of *Z. tritici* has evolved to overcome some qualitative resistances, our study proceeded testing the most highly resistant wheat cultivars against a panel of modern UK fungal isolates. The screening experiment was conducted under two distinct environmental conditions, ensuring a comprehensive assessment of their resistance capabilities against contemporary strains. Under conditions reproducing the UK environment, both Gemmeiza-12 and Misr-3 exhibited a loss of resistance, as evidenced by loss of GLA and significant sporulation. However, it was observed that the necrosis development on Gemmeiza-12 was 52%, 45%, and 9% slower than on Misr-3 at 13 dpi (Fig. 2a,b). These results indicate that among the Egyptian wheat cultivars, Gemmeiza-12 exhibited the highest resistance to these UK isolates. In the second screening experiment, we reduced the humidity to 50% to simulate the environmental conditions observed in certain wheat-growing regions in Egypt. Previous studies have emphasised the significance of high humidity conditions for successful *Z. tritici* infection in wheat [48, 49]. At reduced humidity, the UK isolates still managed to infect both Gemmeiza-12 and Misr-3. However, under the Egyptian-like conditions, disease development was noticeably slower than under UK-like conditions. This infection led to the development of necrosis symptoms, resulting in a decrease in the GLA percentage. However, as a consequence of decreased humidity, the UK isolates all failed to sporulate on Gemmeiza-12 and the KWS-Extase control. Based on these findings, we suggest that the alteration in humidity aided Gemmeiza-12 in preventing pycnidia formation, while it had less impact on the occurrence of necrosis. This is encouraging, as it suggests that infections of Gemmeiza-12 under field conditions may result in limited fungal sporulation and would be unlikely to result in a disease epidemic over the course of a growing season. The modern UK isolates failed to infect the Egyptian durum wheat cultivar Benisuif-7 under either environmental condition. This is consistent with the specificity for hexaploid wheat demonstrated by *Z. tritici* strains recovered from bread wheat [37].

More than twenty qualitative *Stb* resistance genes have been identified in wheat [26]. One of these genes, *Stb6*, is known for conferring specific resistance to IPO323 [25]. To explore the role of *Stb6* in the resistance of the Egyptian wheat cultivars against IPO323, we employed the KASP assay to determine the presence of the resistant allele of *Stb6*. The results revealed that among the tested Egyptian cultivars, eleven possess *Stb6* resistant alleles, while only three cultivars have the *Stb6* susceptible allele (Table 1). Interestingly, the remaining five cultivars displayed none of the tested haplotypes, suggesting that these cultivars either lack *Stb6* or may carry different *Stb6* haplotypes not previously described. Stb6 recognises avirulent alleles of the effector encoded by *AvrStb6* [50] in a gene-for-gene manner, leading to the induction of an effective immune response [25]. The resistance observed in the Egyptian wheat cultivars against IPO323 was lost when infected with an IPO323 mutant lacking the *AvrStb6* gene (Fig. 4). These findings provide conclusive evidence for the contributory role of *Stb6* in conferring resistance to *Z. tritici* in some Egyptian wheat. However, *Stb6* offers limited protection in the field, as most *Z. tritici* isolates now carry virulent alleles of *AvrStb6* [51]. It will therefore be important to introduce other *Stb* genes into Egyptian germplasm in any dedicated breeding program.

As five of the Egyptian wheat cultivars exhibited robust resistance against both IPO323 and IPO88004, we further explored other potential sources of resistance within these cultivars by investigating the presence of other *Stb* genes. For this purpose, standard PCR was used to examine the presence of the resistant allele of *Stb16q*. Our findings revealed that none of the five cultivars that demonstrated resistance to both IPO323 and IPO88004 were found to have the resistant allele of *Stb16q*. A previous study identified another *Stb* gene, *Stb15*, in the Swiss wheat cultivar Arina and Riband [52]. *Stb15* was later found to confer specific resistance against IPO88004. However, the KASP genotyping assay results revealed that none of the Egyptian wheat cultivars possesses the resistant allele of *Stb15* (Haplotype Arina). Hence, we can infer that the resistance observed in the five Egyptian cultivars, namely Gemmeiza-12, Misr-1, Misr-3, Giza-171, and Benisuif-7, may be attributed to other resistance genes. These findings contribute to a better understanding of the diverse sources of resistance within the Egyptian wheat cultivars.

The initial screening showed that the majority of the Egyptian cultivars were resistant to IPO323. To characterise the resistance of the most highly resistant cultivar, we examined the capability of IPO323 spores to adhere to the leaf surface of Gemmeiza-12. The results of this experiment demonstrated that IPO323 spores exhibited a lower ability to adhere to the leaf surface of Gemmeiza-12 compared to Cadenza and Riband (Fig. 5). Previous studies have discussed the role of pycnidiospores of *Z. tritici* in producing cutinases [53]. These enzymes are responsible for breaking down the wax present on wheat leaves and may play a significant role in the initial adhesion of spores [53, 54]. Furthermore, the leaf architecture might also contribute partially to spore adhesion. In a previous study where spore adhesion was evaluated through leaf washing, it was observed that trichomes assisted in *Z. tritici* spore adhesion [55]. Based on these findings, it is possible that the reduced adhesion of IPO323 on the leaf surface of Gemmeiza-12 is due to a modified wax composition and/or fewer trichomes compared to Cadenza and Riband. However, this requires further investigation. We continued to investigate the epiphytic growth of IPO323 on the leaf surface of the three tested cultivars (Fig. 5). Up to 3 dpi, there were minimal differences observed in the epiphytic growth of IPO323 on the leaf surface of the susceptible cultivar Riband, as compared to the resistant cultivars Cadenza and Gemmeiza-12. By 5 dpi, there was no significant difference in the extent of IPO323 hyphal growth on Gemmeiza-12 compared to Riband. This finding agrees with a recent study that *Z. tritici* isolates with different virulence profiles are capable of germinating and growing epiphytically on the leaf surface of wheat [56]. Indeed, *Z. tritici* is also capable of germinating and extending hyphae on leaves of the distantly-related model dicot *Nicotiana benthamiana* [57]. However, the extent of IPO323 hyphal growth on Riband was significantly larger than that observed on Cadenza. It is worth noting that leaves exude nutrients, such as sugars [58], The presence of nutrients on the phyllosphere of Riband might facilitate greater hyphal growth of IPO323 compared to Cadenza.

We also investigated IPO323’s ability to invade the stomata and colonize the sub-stomatal cavity of the three tested cultivars. It is known that *Z. tritici* hyphae attempt to invade the stomata from 2 to 13 dpi, peaking at 4 dpi [59]. We observed that by 5 dpi, hyphae were interacting with stomata of all cultivars examined. However, by 9 dpi, hyphae could only be observed within stomata of the susceptible cultivar Riband and colonising the apoplastic space. By 12 dpi, hyphae filled the sub-stomatal cavity of Riband, forming the fruiting body and this did not occur on Cadenza or Gemmeiza-12. A previous study suggested an association between the immune responses of wheat to *Z. tritici* and the subsidiary cells, which regulate the aperture of stomatal pores [60]. They found that in response to IPO323 infection, Cadenza formed pigmented papilla-like structures, whereas Riband formed less extensive non-pigmented papilla-like structures. Our results indicated that Gemmeiza-12 and Cadenza responded similarly to IPO323. This similarity suggests a shared resistance mechanism, specifically the inability of the fungus to pass guard cells. In a previous study, avirulent isolates were predominantly hindered during their stomatal penetration into wheat tissues by *Stb16q*-mediated resistance. The study revealed that *Stb16q* induces a temporary closure of stomata in response to avirulent isolates. Moreover, both *Stb6* and *Stb9* induce similar reactions to *Stb16q*, suggesting that an arrest during stomatal penetration is a shared mechanism underlying *Stb*-mediated resistance [61].

## Conclusions

The Egyptian wheat cultivars exhibited a broad spectrum of responses to *Z. tritici*. The resistant allele of *Stb6* was identified in the majority of Egyptian cultivars. Among them, five bread wheat cultivars displayed robust resistance to the IPO323 and IPO88004 isolates. Notably, Gemmeiza-12 emerged as the most resistant Egyptian bread wheat cultivar against modern UK *Z. tritici* isolates. These findings hold significant implications for wheat breeders in Egypt, as they provide insights into enhancing the inherent resistance of Egyptian wheat cultivars against *Z. tritici* and suggest that in the event of STB outbreaks in Egypt, Gemmeiza-12 may have promise for prioritising STB resistance in a dedicated breeding program.

## Materials and methods

### Plant growth conditions

The 18 Egyptian wheat cultivars (Additional file 1) consist of 13 bread wheat cultivars, *Triticum aestivum* L. (Misr-1, Misr-2, Misr-3, Gemmeiza-10, Gemmeiza-11, Gemmeiza-12, Sakha-94, Sakha-95, Sakha-1001, Giza-171, Shandaweel-1, Sids-12, and Sids-14), and five durum wheat cultivars, *Triticum turgidum* ssp. *durum* (Benisuif-5, Benisuif-6, Benisuif-7, Sohag-4, and Sohag-5), all of which were sourced from the Egyptian Agricultural Research Institute. All wheat cultivars were grown in half-tray pots containing Jiffy standard peat free compost at 20 °C under a 16-hour light/8-hour dark cycle with humidity levels exceeding 80%. To simulate Egyptian-like conditions, a separate growth room was used at 20 °C under a 16-hour light/8-hour dark cycle with humidity set at 50%. The wheat plants, aged 21 days, were prepared for fungal inoculation by aligning their second leaves on horizontal foam platforms measuring 23 cm x 11.5 cm x 6 cm 24 h before inoculation.

### Fungal preparation and inoculation

*Z. tritici* isolates IPO323 and IPO88004 have been described previously [52]. Thirty UK *Z. tritici* modern isolates were collected from multiple fields in the Kenilworth Castle area of Warwickshire, United Kingdom, in 2021. These isolates were then grown on Potato dextrose agar (PDA) plates and incubated at 18 °C for 7 days. The three selected modern UK isolates (KC_F1_L3_1, KC_F2_L2_5, and KC_F2_L6_3) were collected from two different fields (Coordinates:52.348865°N, 1.618832°W; 52.348865°N, 1.618832°W). The IPO323_CZtGFP reporter strain was generated by Kilaru and colleagues [62]. All *Z. tritici* isolates were cultured on yeast peptone dextrose agar (YPDA) plates at 18 °C for 6 days. To prepare inoculum, pycnidiospores were resuspended in water +(0.01% Tween-20) to a density of 1 × 10^6^ spores/ml. Cotton swabs were used to inoculate the adaxial surfaces of the second leaves of plants, whereas the control plants (Mock) were treated with 0.01% Tween-20 solution. Following inoculation, plants were placed in trays and covered with propagator lids for 72 h to provide 100% humidity for the inoculated leaves. At 3 dpi, the lids were removed to restore the normal humidity level in the growth room, which was set to 80%.

### Disease scoring

Measurement of green leaf area (GLA) was performed by capturing images at 11, 14, 18, and 21 dpi and analysing them with MIPAR® image analysis software [63]. GLA was calculated by measuring the area of the green, chlorotic, and necrotic patches in pixels and determining the percentage of the green leaf area out of the total leaf area. Spore wash counts were performed following the method outlined by Lee and coworkers Lee et al. (2015) [64]. Briefly, at 21 dpi, we detached ∼ 8 cm fungal-inoculated leaf segments. Two ∼ 8 cm segments from each plant were placed in sealed tubes containing 1 ml of sterile water. After overnight incubation at 18 °C, tubes were vortexed for 30 s to wash off pycnidiospores. Spore counts were done using a haemocytometer. Eight independent replicates per cultivar were used for the initial screening of Egyptian cultivars against IPO323 and IPO88004. Six independent replicates per cultivar were used for screening Egyptian cultivars against modern UK isolates. This experiment was replicated twice.

### Genomic DNA extraction from wheat leaves

The DNA extraction from wheat leaf tissue was conducted according to the method described previously [65], with some exceptions. Approximately 25 mg of leaf tissue was collected from 18 Egyptian wheat cultivars, including Cadenza, Riband, KWS-Extase, and two Chinese spring near-isogenic lines (NILs). Each wheat cultivar’s leaf tissue sample was then transferred into 2 ml tubes containing two 3 mm metal beads. The samples were ground into a fine powder using liquid nitrogen and a TissueLyser II (Qiagen, Germany) for 30 s at a frequency of 30 Hz/s. Finally, the DNA quantity and quality were assessed using a NanoPhotometer N50 (IMPLEN, Germany) and gel electrophoresis, respectively.

### Kompetitive allele-specific PCR (KASP) genotyping assay

For each of the 20 wheat cultivars, three plants were grown, and three leaf samples were collected. Each leaf sample was obtained from an individual plant of the respective wheat cultivar. Genomic DNA extraction was performed from each sample using CTAB. To conduct the KASP genotyping assay to investigate the presence of *Stb6*, two KASP markers, cfn80047 and cfn80050, were utilised (Additional file 5). These markers were developed based on single nucleotide polymorphisms (SNPs) located at position 823 and position 1340 on the coding DNA sequence (CDS), respectively. These markers were specifically designed to detect three distinct Stb6 haplotypes. Haplotype 1, denoted as CT, represents the *Stb6* resistant allele. Haplotypes 3 and 7, represented by CA and AT, respectively, signify the *Stb6* susceptible allele. To investigate the presence of *Stb15* in Egyptian wheat, the KASP marker cfn80111 [35] (Additional file 5) was employed to identify two *Stb15* haplotypes: the haplotype Arina, representing the resistance allele in the Arina wheat cultivar, and the haplotype Cs, representing the susceptible allele in the Chinese spring wheat cultivar.

Reactions were performed following instructions provided by the manufacturer (LGC Genomics®). The run cycle was performed as follows: (1) Denaturation stage: The reaction was incubated at 94 °C for 15 min. (2) PCR cycle 1 (10x): Each cycle consisted of denaturation at 94 °C for 20 s, followed by annealing at 65 °C with a decrement of 0.8 °C per cycle for 1 min. (3) PCR Cycle 2 (30x): Each cycle comprised denaturation at 94 °C for 20 s, followed by annealing at 57 °C for 1 min. 3. Post-read stage: The reaction was held at 30 °C for 1 min. The three samples of each cultivar were used as replicates in the experiment.

### Standard PCR

To examine the presence of the resistant allele of *Stb16q* we utilised the previously described PCR primers 20F6/20R7 [66] (Additional file 5). Additionally, two NILs derived from Chinese spring wheat, where one possesses the Stb16q resistant allele and the other does not, were employed as control samples. The PCR cycling conditions were as follows: (1) Denaturation stage: 95 °C for 30 s. (2) PCR Cycle (30x): Each cycle consisted of 94 °C for 15 s, 64 °C for 30 s, 68 °C for 58 s. Extension stage: 68 °C for 5 min. The DNA fragments were visualized using a 1.2% agarose gel.

### Spore adhesion assay

The second leaves of 21-day-old wheat plants were attached to foam platforms to achieve an approximate inclination angle of 48 degrees. For each wheat cultivar, five plants were selected as replicates and inoculated with IPO323_CZtGFP by spraying with *Z. tritici* spore suspension (1 × 10^6^ spores/ml). Leaves were continuously sprayed with inoculum until runoff. The inoculated plants were covered with lids for 24 h to create 100% humidity conditions. At 24 hpi, three leaf segments, each with a surface area of 50 mm^2^, were harvested from each of the five replicates per cultivar, resulting in a total of 15 leaf segments per cultivar. The first 2 cm from the leaf edges were removed. Each leaf segment was then transferred to a 2 ml microtube containing 1 ml of a 0.01% Tween-20 solution and one 3 mm metal bead. All the leaf segments were homogenised using a TissueLyser II (Qiagen, Germany) at a frequency of 30 Hz/s for 30 s. Following homogenisation, the solution in each tube was diluted 1:4, and 50 µl of the diluted solution was plated on YPDA media supplemented with 40 µg/ml carboxin. The number of spores that adhered was determined by counting the colony forming units (CFU) after 8 days of incubation at 18 °C. This experiment was replicated twice.

### Epiphytic fungal growth and stomatal invasion assay

This experiment was conducted according to [59] with some modifications. Gemmeiza-12, Riband, and Cadenza were inoculated with IPO323_CZtGFP using cotton swab. Leaf samples were collected immediately following inoculation (0 dpi), 3 dpi, and 5 dpi. At each time point, three replicates of leaf samples measuring ∼ 1 cm in length were taken from three different plants of each cultivar. Leaf samples were placed on glass slides with a single drop of 60% glycerol. GFP fluorescence was observed using a Zeiss LSM-780 laser scanning confocal microscope (Carl Zeiss Microscopy, Germany) equipped with a 10x objective lens and Hyd detectors. The microscope was set to standard mode with an excitation wavelength range of 495–525 nm. Five images were randomly captured from different locations on each leaf sample, resulting in a total of 15 images per cultivar at each time point. All the images were analysed using ImageJ to determine the average size of the spores (and associated filamentous growth) in pixels on each cultivar at each time point.

To investigate stomatal penetration, leaf samples measuring ∼ 1 cm in length were collected as described above at time points: 5 dpi, 9 dpi, and 12 dpi. GFP fluorescence was observed as above, and the fluorescence emitted by chloroplasts was detected using a second Hyd detector set to a wavelength range of 700–790 nm. To induce autofluorescence in the plant tissue, a 405 nm laser at 20% output power was used, and the emitted fluorescence was captured using a PMT detector within the range of 411–486 nm. Z-stack Images were taken as described above, using a 40x objective upon the recognition of GFP fluorescence. The fungal GFP signal was quantified using ImageJ software as % of total signal. Three independent replicates were used for each cultivar.

### Electronic supplementary material

Below is the link to the electronic supplementary material.

Supplementary Material 1

Supplementary Material 2

Supplementary Material 3

Supplementary Material 4

Supplementary Material 5

Supplementary Material 6

## Data Availability

All data sheets and images are available upon request to the corresponding author, Graeme J Kettles (g.j.kettles@bham.ac.uk).

## Abbreviations

STB: Septoria tritici blotch
MMT: Million metric tonnes
QTL: Quantitative trait loci
WAK: Wall-associated receptor kinase
GLA: Measurement of green leaf area
YODA: yeast peptone dextrose agar
KASP: Kompetitive allele-specific PCR
CDS: Coding DNA sequence
SNP: Single nucleotide polymorphisms
CFU: Colony forming units
GFP: Green fluorescent protein
DPI: Days post inoculation
NILs: Near-isogenic lines
