# Supplementary material for: The Egyptian wheat cultivar Gemmeiza-12 is a source of resistance against the fungus Zymoseptoria tritici
Source: BMC Plant Biol. 2024 Apr 5;24:248. doi: 10.1186/s12870-024-04930-y (PMC10996218; doi:10.1186/s12870-024-04930-y)
Supplement: Supplementary file 1 — Supplementary Material 1 [file 12870_2024_4930_MOESM1_ESM.docx]

**Additional file 5. Primer sequences for STB resistance genes.**

| **Primer names** | **Sequences** | **Comments** |
| --- | --- | --- |
| 20F6 | CACGGCGGCTTCCTTATTGTA | Screening for *Stb16q* resistant allele |
| 20R7 | TGGGGCAATTATGTGGCAAC | Screening for *Stb16q* resistant allele |
| cfn80047_F | GAAGGTGACCAAGTTCATGCTCTGCAACCTTTCTCTTTGCATGTC | *Stb6* diagnostic marker |
| cfn80047_H | GAAGGTCGGAGTCAACGGATTCTGCAACCTTTCTCTTTGCATGTA | *Stb6* diagnostic marker |
| cfn80047_C | GGAAAAACCATAGTCCTTTCCCATT | *Stb6* diagnostic marker |
| cfn80050_F | GAAGGTGACCAAGTTCATGCTgggtttgatgtcgaaatggatga | *Stb6* diagnostic marker |
| cfn80050_H | GAAGGTCGGAGTCAACGGATTgggtttgatgtcgaaatggatgt | *Stb6* diagnostic marker |
| cfn80050_C | GGATAAGTACATTTACTCAGGGAGCC | *Stb6* diagnostic marker |
| cfn80111_F | GAAGGTGACCAAGTTCATGCTGGTTTCAACTTGCAATATGATC | *Stb15* diagnostic marker |
| cfn80111_V | GAAGGTCGGAGTCAACGGATTGGTTTCAACTTGCCATATGATT | *Stb15* diagnostic marker |
| cfn80111_C | AGTGAACCAGGTGCCAAAAC | *Stb15* diagnostic marker |
