# Supplementary material for: The Egyptian wheat cultivar Gemmeiza-12 is a source of resistance against the fungus Zymoseptoria tritici
Source: BMC Plant Biol. 2024 Apr 5;24:248. doi: 10.1186/s12870-024-04930-y (PMC10996218; doi:10.1186/s12870-024-04930-y)
Supplement: Supplementary file 2 — Supplementary Material 2 [file 12870_2024_4930_MOESM2_ESM.docx]

**Additional file 3. KASP diagnostic assay to determine presence of the *Stb15* resistance allele in Egyptian wheat cultivars.**

| **Wheat cultivar** | **Haplotype Arina (Stb15)** | **Haplotype Cs** |
| --- | --- | --- |
| Cadenza |  | * |
| Riband | * |  |
| KWS-Extase | * |  |
| Benisuif-5 |  | * |
| Benisuif-6 |  | * |
| Benisuif-7 |  | * |
| Sohag-4 |  | * |
| Sohag-5 |  | * |
| Misr-1 |  | * |
| Misr-2 |  | * |
| Misr-3 |  | * |
| Gemmeiza-10 |  | * |
| Gemmeiza-11 |  | * |
| Gemmeiza-12 |  | * |
| Sakha-94 |  | * |
| Sakha-95 |  | * |
| Sakha-1001 |  | * |
| Giza-171 |  | * |
| Shandaweel-1 |  | * |
| Sids-12 |  | * |
| Sids-14 |  | * |

The resistant allele of *Stb15* is represented by the Arina haplotype. The susceptible allele is represented by the Cs haplotype. Cadenza, Riband, and KWS-Extase were used as controls.
