# Supplementary material for: The Egyptian wheat cultivar Gemmeiza-12 is a source of resistance against the fungus Zymoseptoria tritici
Source: BMC Plant Biol. 2024 Apr 5;24:248. doi: 10.1186/s12870-024-04930-y (PMC10996218; doi:10.1186/s12870-024-04930-y)
Supplement: Supplementary file 3 — Supplementary Material 3 [file 12870_2024_4930_MOESM3_ESM.docx]

**Additional file 1. List of Egyptian wheat *c*ultivars investigated in the *s*tudy.**

| **Wheat cultivar** | **Year of release** | | **Growing area in Fadden** | **% of Egypt’s total wheat-growing area** |
| --- | --- | --- | --- | --- |
| Benisuif-5 | 2008 | | 390312 | 11.415 |
| Benisuif-6 | 2009 | | 4445 | 0.130 |
| Benisuif-7 | 2017 | | 2 | 0.000 |
| Sohag-4 | 2014 | | 10084 | 0.295 |
| Sohag-5 | 2016 | | 311 | 0.009 |
| Misr-1 | 2009 | | 727793 | 21.284 |
| Misr-2 | 2010 | | 202292 | 5.916 |
| Misr-3 | 2016 | | 2132 | 0.062 |
| Gemmeiza-10 | 2004 | | 34 | 0.001 |
| Gemmeiza-11 | 2010 | | 205563 | 6.012 |
| Gemmeiza-12 | 2012 | | 46457 | 1.359 |
| Sakha-94 | 2004 | | 10496 | 0.307 |
| Sakha-95 | 2016 | | 20863 | 0.610 |
| Sakha-1001 | 2020 | | - | - |
| Giza-171 | 2012 | | 779316 | 22.791 |
| Shandaweel-1 | 2013 | | 10607 | 0.310 |
| Sids-12 | 2011 | | 223062 | 6.523 |
| Sids-14 | 2017 | | 348118 | 10.181 |
| **Total** | | **-** | **2981887** | **87.204** |
