# Supplementary material for: The Egyptian wheat cultivar Gemmeiza-12 is a source of resistance against the fungus Zymoseptoria tritici
Source: BMC Plant Biol. 2024 Apr 5;24:248. doi: 10.1186/s12870-024-04930-y (PMC10996218; doi:10.1186/s12870-024-04930-y)
Supplement: Supplementary file 5 — Supplementary Material 5 [file 12870_2024_4930_MOESM5_ESM.docx]

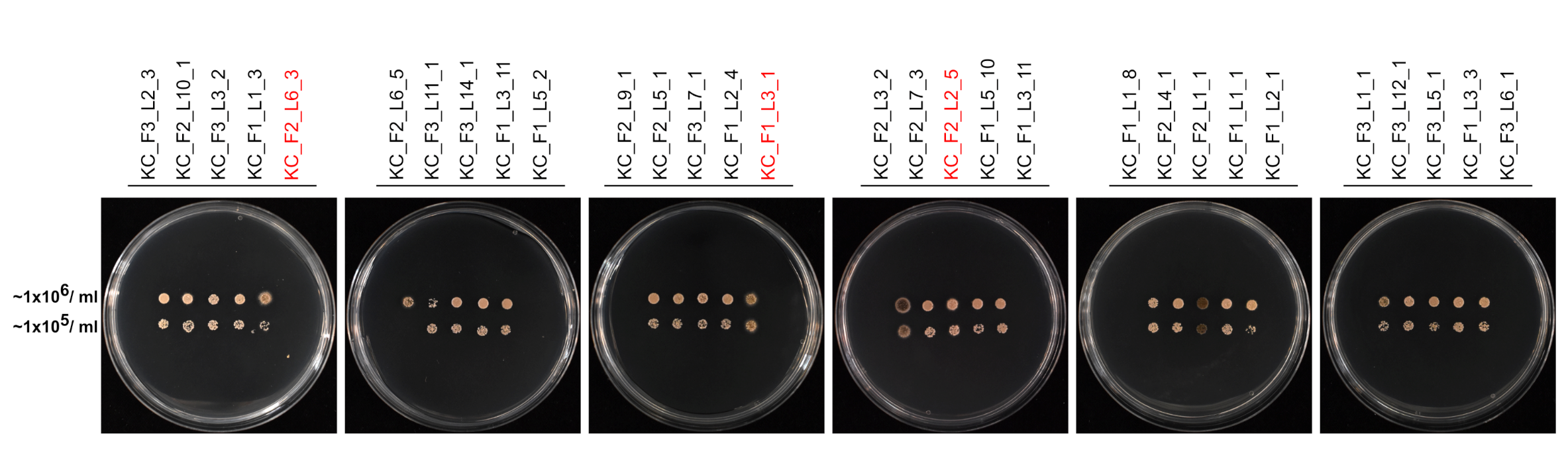


**Additional file 2. The phenotypes of a panel of 30 modern UK *Z. tritici* isolates on PDA plates.** The spores of each isolate were applied on the PDA plates in two concentrations ~1x10^6^ and ~1x10^5^. The selected isolates are highlighted in red.
