# Supplementary material for: The Egyptian wheat cultivar Gemmeiza-12 is a source of resistance against the fungus Zymoseptoria tritici
Source: BMC Plant Biol. 2024 Apr 5;24:248. doi: 10.1186/s12870-024-04930-y (PMC10996218; doi:10.1186/s12870-024-04930-y)
Supplement: Supplementary file 6 — Supplementary Material 6 [file 12870_2024_4930_MOESM6_ESM.docx]

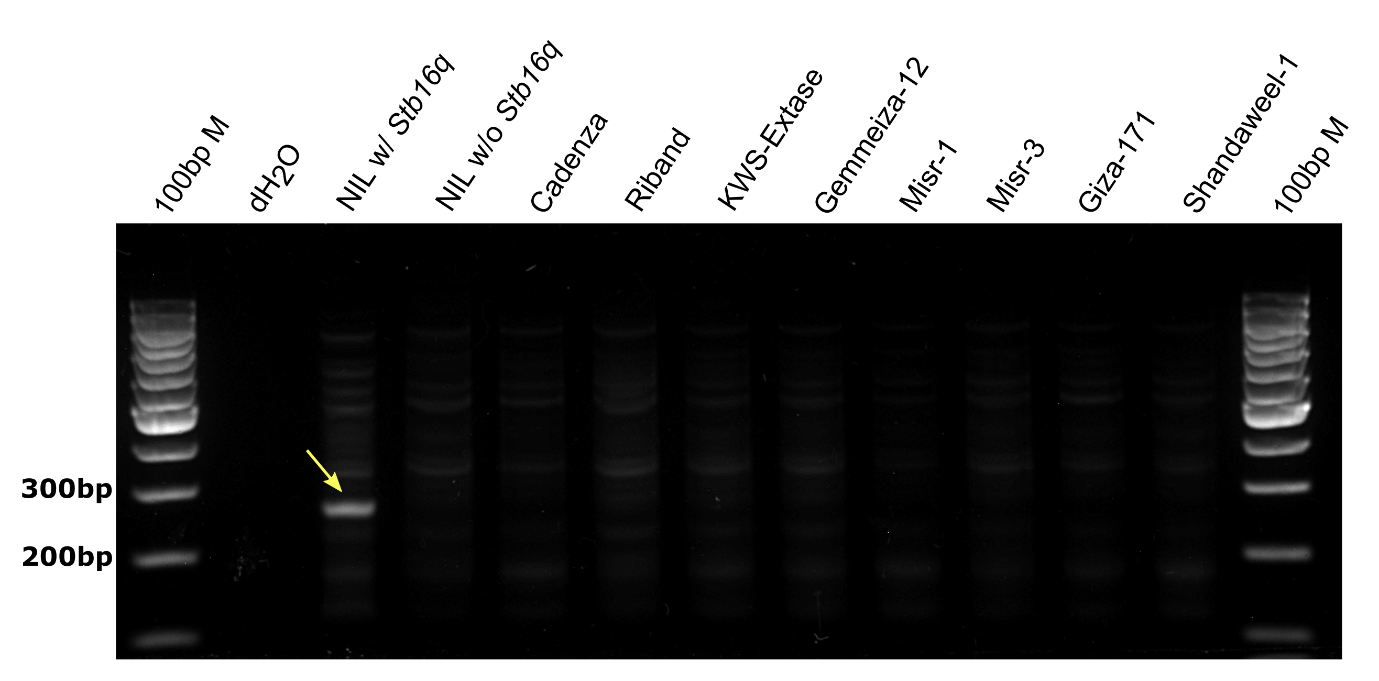


**Additional file 4. The *Stb16q* resistance gene is not present in Egyptian wheat.** Two Chinese spring wheat NILs were used as controls. One NIL has the resistance allele of the *Stb16q* gene, while the other NIL lacks the resistance allele of *Stb16q*. The yellow arrow points to the amplified band of the *Stb16q* resistance allele.
